# Generative Adversarial Networks in Digital Pathology: A Survey on Trends and Future Potential

**DOI:** 10.1016/j.patter.2020.100089

**Published:** 2020-09-11

**Authors:** Maximilian E. Tschuchnig, Gertie J. Oostingh, Michael Gadermayr

**Affiliations:** 1 Department of Information Technologies and Systems Management, Salzburg University of Applied Sciences, 5412 Puch bei Hallein, Austria; 2 Department of Biomedical Sciences, Salzburg University of Applied Sciences, 5412 Puch bei Hallein, Austria

**Keywords:** generative adversarial network, computational pathology, histology, image-to-image translation, survey

## Abstract

Image analysis in the field of digital pathology has recently gained increased popularity. The use of high-quality whole-slide scanners enables the fast acquisition of large amounts of image data, showing extensive context and microscopic detail at the same time. Simultaneously, novel machine-learning algorithms have boosted the performance of image analysis approaches. In this paper, we focus on a particularly powerful class of architectures, the so-called generative adversarial networks (GANs) applied to histological image data. Besides improving performance, GANs also enable previously intractable application scenarios in this field. However, GANs could exhibit a potential for introducing bias. Hereby, we summarize the recent state-of-the-art developments in a generalizing notation, present the main applications of GANs, and give an outlook of some chosen promising approaches and their possible future applications. In addition, we identify currently unavailable methods with potential for future applications.

## The Bigger Picture

The use of high-quality whole-slide scanners enables the fast acquisition of large amounts of image data, showing extensive context and microscopic detail at the same time. While manual examination of these images of considerable size is highly time consuming and error prone, state-of-the-art machine-learning approaches enable efficient, automated processing of whole-slide images. In this paper, we focus on a particularly powerful class of deep-learning architectures, the so-called generative adversarial networks. Over the past years, the high number of publications on this topic indicates a very high potential of generative adversarial networks in the field of digital pathology. In this survey, the most important publications are collected and categorized according to the techniques used and the aspired application scenario. We identify the main ideas and provide an outlook into the future.

## Teaser

Whole-slide scanners digitize microscopic tissue slides and thereby generate a large amount of digital image material. This advocates for methods facilitating (semi-)automated analysis. In this paper, we investigate generative adversarial networks, which are a powerful class of deep-learning-based approaches, useful in, for example, histological image analysis. The most important publications in the field of digital pathology are collected, summarized, and categorized according to the technical approaches employed and the aspired application scenarios. We identify the main findings and furthermore provide an outlook.

## Motivation

Whole-slide scanners are capable of effectively digitizing histological or cytological slides without any significant manual effort. These scanners are capable of generating vast amounts of digital data, since a single whole-slide image can show up to several gigapixels in resolution. Digitization opens up the potential for more effective storage, as well as optimized and standardized visualization and transmission (telepathology). However, to outweigh additional effort and thereby make digitization in pathology attractive for routine use, tools for computer-aided analysis are indispensable. Automated methods can provide support by facilitating basic routine tasks, such as counting objects or segmenting regions. Moreover, state-of-the-art machine-learning approaches exhibit a potential for recognizing patterns that normally cannot be easily detected, even by the trained human eye.^1^ Therefore, especially for less experienced pathologists, machine-learning approaches exhibit high potential not only to decrease the time needed but also to improve the diagnostic accuracy. A further motivation is provided by considerable inter-rater variability in histological examinations.^2^^,^^3^

Microscopic evaluation of tissues or cytological preparations is the gold standard in clinical diagnostics for a large range of pathologies. Examples of these are smear tests, analysis of the borders of cancerous tissues during operations, and postmortem histological testing. Due to an increasing prevalence and, thus, workload in the field of cancer-related diseases in combination with a decrease in the number of pathologists,^4^^,^^5^ automated assistance tools will be of major importance in the near future. By facilitating effective automated computer-based processing, image analysis approaches can be a powerful tool to aid clinical practice. Apart from supporting the pathologists' daily routine, automated high-throughput processing techniques can be employed to boost the potential of histological research regarding medical and biological data.

### Image Analysis in Digital Pathology

A particularly relevant application field of digital pathology is exemplified by the detection of tissues of interest combined with a pixel-accurate segmentation. Tasks such as nucleus,^6^, ^7^, ^8^ cancer,^9^^,^^10^ and gland^11^^,^^12^ segmentation have been considered in recent studies. Segmentation approaches combined with the extraction of features, such as quantity, area, and morphological characteristics, allow for the access to image information in an efficient and condensed manner. Classification approaches^13^^,^^14^ go one step further and have the potential to provide an observer-independent decision. While such approaches are completely automated and observer independent, an open issue in practice is how to deal with these black-box decisions when the estimated performance measure (e.g., F-score) does not indicate a perfect categorization (even if an algorithm is as accurate as a human expert). Stain normalization^15^, ^16^, ^17^ also represents an important field, allowing for the harmonization of data from a single or several different image modalities showing stain variability. Stain normalization can be used as pre-processing for computer-based analysis and to enhance manual experts' examination performance. For training automated image analysis models, stain augmentation (by simulating a wide variety of realistic stain variations) is an alternative to stain normalization. Recent research has shown that a combination of both exhibits the best performance.^18^

From a technical point of view, a wide range of different approaches have been applied to histological image data. Before the era of deep learning, pipelines especially based on thresholding,^19^ watershed,^20^ active contours,^21^ and a combination of these approaches were proposed for the purpose of segmentation. Stain-normalization approaches were mainly based on pixel-level transformations^15^^,^^17^ such as color deconvolution.^15^ Pixel level in this context means that mappings are generated without incorporating the pixel neighborhood. Classification approaches were based on separate feature extraction (e.g., local binary patterns,^22^ Fisher vectors^23^) and classification models such as support vector machines.^24^

Recently, deep-learning approaches and particularly convolutional neural networks (CNNs) have been identified as highly powerful and generic tools, being capable of performing a large range of tasks.^25^^,^^26^ In many application scenarios, deep-learning methods outperformed the existing approaches.^25^ Especially in the field of segmentation, the so-called fully convolutional networks using skip-connection,^12^^,^^27^^,^^28^ such as the prominent U-Net,^27^ boosted segmentation accuracy and exhibited high efficiency. This allowed for rapid processing of huge images in combination with relatively inexpensive consumer graphics processing units.

### Challenges

A disadvantage, however, of many deep-learning approaches lies in the fact that these methods typically need large amounts of labeled training data. Data augmentation can be a powerful tool to lessen this restriction.^27^^,^^29^^,^^30^ Nevertheless, a significant amount of manually annotated data is mostly indispensable. Due to the large image size of up to several gigapixels, manual annotation of histological whole-slide images for the purpose of segmentation can be extremely time consuming. As this task often needs to be performed by medical experts, this fact constitutes a burden for the application of deep-learning approaches in practice. A further difficulty arises due to the variability in the image domain,^17^ which is typically (unintentionally) caused by differences in the cutting and staining process. Intentional differences can also be due to other staining techniques, applied to extract other or additional features from the image data. Varying staining techniques showing similar morphologies, but different texture and color characteristics, also require individually trained image analysis models. A further source of variation is given by intra-subject variability, for example due to (a wide range of) different pathologies. The use of a standard deep-learning pipeline (without domain adaptation) advocates for manually annotated training data. These training data have to cover the whole range of image characteristics, which can be extremely diverse if several degrees of variation occur.^31^

Approaches relying on generative adversarial networks (GANs)^32^ exhibit the potential to reduce the requirement of large amounts of manual annotations. This, in turn, reduces the barrier to entry for automated image analysis methods in medical imaging. Particularly in the field of digital pathology, recent developments not only improved measures but even enabled novel applications. Many tasks for which supervised learning approaches were indispensable can now be performed with unsupervised techniques.

### Contribution

In this review, we summarize the application scenarios and recent developments of GAN-based approaches in the field of digital pathology. Based on this research, we highlight application scenarios that clearly profit from recent GAN approaches using some of the most prominent architectures and adaptations of these architectures. We also identify remaining issues and challenges and determine relevant highly potential fields of research for the future. Finally, we also provide uniform definitions to facilitate an orientation in the “jungle of GANs.”

First, a summary and classification (based on capabilities) of architectures applied to digital pathology are provided. Next, the histological application scenarios are outlined, followed by a review of the individual approaches. We then discuss trends, benefits, challenges, and additional potential of GANs before concluding the review.

## GAN Architectures

**Figure 1:**
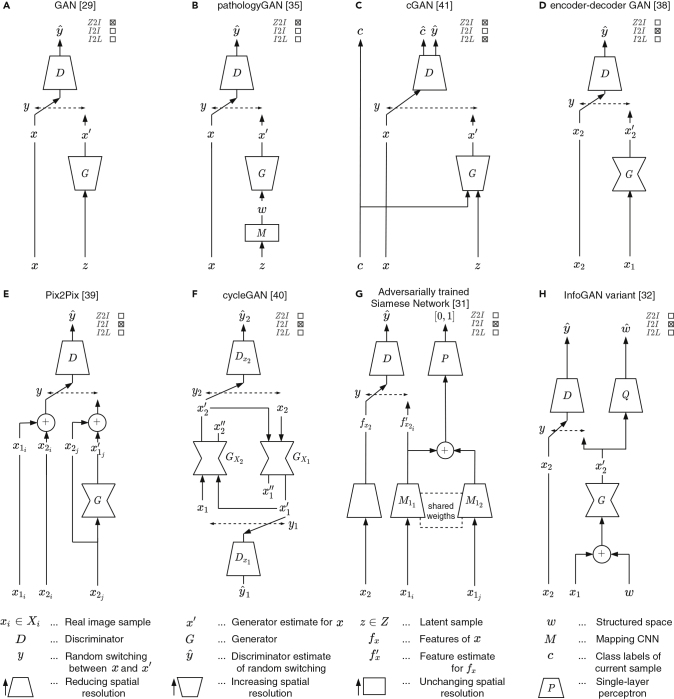
Architecture Comparison of Several Latent-to-Image, Image-to-Image, and Image-to-Label Networks for Digital Pathology, Trained Primarily through an Adversarial Loss

The idea of training two neural networks in a zero-sum min-max game is shown to enable stable training in image analysis for digital pathology with several important architectures such as GAN, cGAN, cycleGAN, InfoGAN, BigGAN and GAN-based Siamese Networks.^8^^,^^33^, ^34^, ^35^, ^36^, ^37^, ^38^, ^39^, ^40^ In this section, we focus on the technical background of GAN approaches employed for image analysis in digital pathology. We analyze these architectures and cluster them into similar groups (Figure 1) with respect to their applicability. Additionally, we summarize the capabilities of individual GAN architectures, and analyze evaluation methods for GAN-based augmented medical images.

The conventional GAN architecture, introduced by Goodfellow et al.,^32^ is shown in Figure 1A. It enables the generation of image data by mapping an unstructured latent space into an image *Z* → *X*, using an up-scaling CNN, called a generator *G*. To generate images showing the desired characteristics, this generator is trained with the aim of fooling a discriminator *D*. The discriminator, typically also a CNN, is trained to distinguish between real (*x* ∈ *X*) and generated samples (*G*(*z*), *z* ∈ *Z*). The labeling *y* describes the data to be real if *y* = 1 and generated if *y* = 0. Therefore, $\hat{y}$ describes the discriminator's prediction, which is used by the so-called adversarial loss $\left(loss\left(y,\hat{y}\right)\right)$.^32^ This loss is incorporated in all of the following GAN architectures.

This basic architecture can be adjusted in multiple ways and for a multitude of tasks. These adjustments are, for example, the addition of a mapping layer before the generator (Figure 1B^38^) or the replacement of the generator with an encoder-decoder structure (Figure 1D^41^). By replacing the generator with an encoder-decoder structure (Figure 1D), image transformations from one domain to another are enabled.^42^ This can be achieved by replacing the latent space *Z* with images of the source domain *X*_1_ and training the generator to transform *X*_1_ into a realistic image of the domain *X*_2_ using an adversarial loss. This enables applications such as segmentation (*X*_1_ → *X*_2_). Adding a mapping network between *Z* and the generator (Figure 1B) aims at mapping the unknown latent space *Z* to a structured latent space *W*. This interpretable structure enables semantic vector operations that translate into domain-specific feature transformations.^38^

cGAN (Figure 1C), in comparison with the original GAN approach (Figure 1A), adds class information (c). The discriminator distinguishes between real (*x*) and generated (*G*(*z*|*c*)) samples with the class as an additional condition. This is obtained by adding a weighted classification loss $\left(loss\left(c,\hat{c}\right)\right)$ to the adversarial loss. This architecture is capable of constructing images from different classes based on a single generator.^43^

Pix2Pix^42^ (Figure 1E) is a variation of cGAN^40^ that replaces the up-scaling generator for an encoder-decoder structure and the class information with the corresponding image from the second domain. Therefore, the Pix2Pix generator learns to translate between two image domains (*X*_1_ → *X*_2_). For training, Pix2Pix needs corresponding samples (pairs) showing images from the two domains capturing the same underlying content. Therefore, the requirements are the same as for (fully convolutional) segmentation networks, such as U-Net.^27^ To train Pix2Pix, a combination of the adversarial loss $\left(loss\left(y,\hat{y}\right)\right)$ and the $L_{2}\left(y,\hat{y}\right)$ loss is optimized. Since the input data are a pair, the adversarial loss analyzes if the conversion *X*_1_ → *X*_2_ (and not only the output) is realistic.

cycleGAN^44^ is an approach (Figure 1F) that enables unpaired image translation through an adversarial loss in combination with a cycle-consistency loss. The core idea is to train two generators to transfer images from domain *X*_1_ to domain *X*_2_ and vice versa. Therefore, a loss can be calculated by combining the adversarial loss $\left(loss\left(y_{1},\hat{y_{1}}\right)\right)$ with a cycle-consistency loss $\left(loss\left(x_{1},{x_{1}}^{\prime \prime }\right)\right)$, with ${x_{1}}^{\prime \prime }$being $G_{X_{1}}\circ G_{X_{2}}\left(x_{1}\right)$ for both image domains. This cycle-consistency loss penalizes changes in structural information from the real to the reconstructed sample.^44^ Without further constraint, the generators typically also maintain the structure in the virtual domain. This is probably because a significant modification of the underlying structure followed by the inverse modification would be more complex to learn.

Combining the idea of adversarial learning with a Siamese network,^45^ as shown in Figure 1G, results in a feature-based domain-transfer method.^34^ The far-left part of this network structure (*M*_2_) is a CNN that is trained in a supervised manner and encodes the data from domain *X*_2_ in a feature space $f_{x_{2}}$. The CNN $M_{1_{1}}$ is trained adversarially in order to extract realistic features of domain *X*_2_ from domain *X*_1_. To keep the domain information of *X*_1_, the networks $M_{1_{1}}$ and $M_{1_{2}}$ are trained as a Siamese network. As shown by Figure 1, the features from $M_{1_{1}}$ and $M_{1_{2}}$ are concatenated and evaluated by a CNN on how well the domain information is kept. This formulation leads to a loss combining the adversarial loss and a mutual information term (*MI*($M_{1_{1}}$($x_{1_{i}}$),$M_{1_{2}}$($x_{1_{j}}$))).

The InfoGAN variant^46^ introduced in Figure 1H^35^ shows a variation on the idea of pathologyGAN that aims to add structure to the latent space in order to have control over the generator results and the kind of features it produces. The InfoGAN^35^ variation aims at finding a structured latent space (*W*) to decouple the color information from the underlying image information. This is accomplished by initially concatenating the noise matrix with the image sample from the second domain and then applying the result into an encoder-decoder structured generator. This generator is trained adversarially as well as through the mutual information of the auxiliary network $\left(MI\left(w,\hat{w}\right)\right)$ that aims at separating the reconstruction from its structured latent part.

### GAN Capabilities in Image Analysis

For differentiation of the capabilities, we decided to use a generic scheme (as indicated in Figure 1). We defined the applications as mappings from one input domain to another output domain. Particularly, we differentiated between Latent-to-Image (Z2I), Image-to-Image (I2I), and Image-to-Label (I2L) translation. We further identified Image-to-Latent (I2Z) as an extension of I2I.

#### Latent-to-Image

The application corresponds to the original GAN idea of generating images out of noise.^32^ This results in a network that can produce a theoretically infinite number of images based on unstructured latent samples (*z* ∈ *Z*). Such a mapping (Z2I: *Z* → *X*) is typically performed by conventional GANs, cGANs, and their various modifications such as the progressive-growing GAN^47^^,^^48^ and Wasserstein GAN,^49^ partially displayed in Figures 1A, 1B, 1C, and 1H. Additionally, latent samples can be mapped to a structured space before image generation to enable interpretable modifications.^35^^,^^38^

#### Image-to-Image

Another typical application of GANs can be summarized as I2I, i.e., a mapping from one image domain *X*_1_ to another image domain *X*_2_ is learned (I2I: *X*_1_ → *X*_2_). For means of generalization, we explicitly also categorized segmentation mask domains as image domains. We decided on this generalization since the same technical approaches are used for the purpose of I2I, Image-to-Mask (known as segmentation), and Mask-to-Image translation (known as image synthesis). For this purpose, Pix2Pix and cycleGAN (Figures 1E and 1F) as well as further GANs such as the encoder-decoder GAN (Figure 1D) and the adversarially trained Siamese networks (Figure 1G), can be applied. Training of I2I approaches can be categorized into two major classes, namely paired (Pix2Pix and InfoGAN) and unpaired (cycleGAN, encoder-decoder GAN, and the adversarial Siamese network). While paired training requires corresponding samples from the two domains for training, unpaired training only needs two individual datasets from both domains. Paired approaches typically exhibit better performance, whereas unpaired techniques enable additional areas of application, since paired data are not always available.^44^ An extension of I2I is to perform representation learning on the output of the generator. This enables the I2I generators to translate between two domains while also maintaining certain characteristics.^50^ Figure 1H, for example, shows the InfoGAN variant, learning a pre-defined color representation in an I2I setting.^46^

#### Image-to-Label

I2L translation is typically referred to as classification. A network is trained to find a mapping I2L: *X* → {0,1, …,*n*} from an image domain *X* to a label domain comprising *n* classes. For this purpose, cGANs and cGAN variants can be employed (Figure 1C), since the discriminator is also trained to determine the class label.^51^

### Evaluation

In the application of GANs being used to augment or balance training datasets for subsequent classification^34^^,^^37^^,^^41^^,^^50^^,^^52^ or segmentation,^6^^,^^8^^,^^31^^,^^33^^,^^37^^,^^39^^,^^41^^,^^53^, ^54^, ^55^ evaluation is straightforward, since typical metrics like F-score or accuracy for classification can be used. However, if there are no labels regarding a final target, these full-reference metrics are not applicable. A measure circumventing the need for target labels is given by the Fréchet inception distance,^38^^,^^56^ which is an objective metric that is able to compare input image distributions with output image distributions by using the inception v3 model.^57^ Another common method of evaluating augmented and generated images is through Turing tests, whereby human experts are placed as another discriminator of real and fake data.^29^^,^^38^^,^^58^^,^^59^

## Tasks in Digital Pathology

I2I, I2L, and Z2I correspond to a multitude of applications in histological image analysis. Here, we identified applications specific for digital pathology and assigned them to one of these translation settings.

Specifically, we identify in the following subsections stain normalization, stain adaptation, segmentation using supervised models, the synthesis of image data for enabling weakly supervised and unsupervised learning, and data augmentation as I2I translation settings. Data augmentation can be both I2I and Z2I depending on the specific configuration. For disambiguation of the application categories, we refer the reader to Table 1. In the following subsections, publications in the field of digital pathology are categorized into one of these settings corresponding to the main contribution of the GAN approach.

**Table 1:** Disambiguation of Pathological Application Scenarios

Application	Setting	Source Domain	Target Domain
Stain normalization	I2I	image data showing (high) variability	image data with lower variability (potentially subset of source domain); unchanged underlying tissue characteristics
Stain and domain adaptation	I2I	image data acquired with a specific imaging setting (staining, scanner)	imaging setting different from source domain (mainly different staining); unchanged underlying tissue characteristics
Segmentation with supervised models	I2I	image data	label masks corresponding to the input image data
Synthesis enabling weakly supervised and unsupervised learning	I2I	label masks (standard case)	image data corresponding to the label masks
		image data	label masks corresponding to the image data
	I2I and I2Z	label masks	image data corresponding to the label masks and image representations
Data generation and augmentation classification	Z2I	latent vector	image data
	I2L	image data	classification labels

We defined stain normalization as a mapping from an original image domain to a normalized domain showing lower variability. With stain adaptation, we refer to the setting whereby not (only) the variability within one staining protocol, e.g., hematoxylin and eosin (H&E), but between different protocols, need to be compensated. In this subsection we also included domain adaptation, which is a generalization of stain adaptation but not a typical I2I setting. Domain adaptation in general is not necessarily performed on image level but on feature level. Regardless, we decided upon this categorization due to the similarity from the application's point of view. According to our definition, stain adaptation can be interpreted as a special type of domain adaptation. In this paper, data augmentation refers to the I2I setting and data generation refers to the Z2I configuration.

### Stain Normalization

Since stain normalization is a type of I2I translation, several GAN-based approaches, as introduced in the subsection GAN Architectures, can be used to enable stain normalization. cycleGAN (Figure 1F) can be optimized for the means of stain normalization, based on one training dataset from a general (*X*_*o*_) and a normalized domain (*X*_*n*_). Pairs, which are difficult to collect for this scenario, are therefore not needed. De Bel et al.^37^ investigated various experimental settings with different generator architectures combined with data-augmentation strategies for cycleGAN. They showed that stain normalization using the baseline architecture performs well and eliminates the need for any further stain augmentation. It is shown in general that cycleGAN is highly flexible and powerful and exhibits a general-purpose architecture that is capable of stain normalization. However, compared with common pixel-based stain-normalization approaches,^15^^,^^17^ cycleGAN can do more than apply a non-linear pixel-based mapping. The approach is theoretically also able to generate changes in texture. Depending on the used datasets for training, this capability corresponds to the potential of introducing bias. This is especially the case if *X*_*o*_ and *X*_*n*_ show systematic differences regarding the underlying tissue characteristics (e.g., in the case of data showing variable degrees of pathologies). Experiments have proved good performance in general, also with respect to final segmentation or classification tasks.^37^ However, an experimental investigation of the impact of different distributions in the two datasets used for training the model has not been performed so far. To eliminate bias in these kinds of architectures, the stain-normalization stage can be integrated into a classification approach.^41^

Other approaches performing unpaired I2I translation for stain normalization replace the cycle-consistency loss with an alternative formulation. Bentaieb and Hamarneh^41^ used an encoder-decoder GAN (Figure 1D) with an additional loss to keep morphological consistency. This is achieved through a further gradient loss. Additionally, this stain-normalization model is combined with a classification model, which can be used to add a classification loss in order to optimize the separability of the classes. The potential of the additional loss here faces reduced flexibility, since the model needs to be trained individually for each task. Nevertheless the limitation is modest, as classification models are necessarily trained or adapted for each specific task.

Zhou et al.^52^ adapt cycleGAN for stain normalization on a single dataset consisting of multiple input characteristics. The first step to achieve a stain-independent normalized color space is based on cluster analysis, which partitions the dataset into a tight *X*_*n*_ and a loose subset *X*_*o*_. Furthermore, the color information, extracted from *X*_*o*_, is used as an auxiliary input for the generator to restore the original color information. This is supposed to stabilize the GAN cycle in potential one-to-many^60^^,^^61^ mapping situations.

A different stain-normalization approach is based on the idea of InfoGAN (Figure 1H). Zanjani et al.^35^ replaced the latent space *Z* with the lightness channel of the source image. Additionally, a mutual information loss was used to train the generator to represent the structured space *W* as the color transformation of the source image. This allowed for the normalization into a pre-defined structured space or in the case of Zanjani et al.,^35^ a pre-defined color space.

Due to the enormous size of whole-slide images, processing is mostly performed patchwise. This, in combination with instance normalization, can lead to tiling artifacts. It was shown that these artifacts can be clearly reduced by adding an additional perceptual loss.^62^

Even though stain normalization is specific for digital pathology, similar approaches are used for harmonization in related applications such as the normalization of magnetic resonance images.^63^

### Stain and Domain Adaptation

One approach to performing domain as well as stain adaptation is to employ cycleGAN (Figure 1F) as introduced by Huang et al.^64^ These authors performed segmentation based on previously adapted image data. While the adaptation method is based on cycleGAN and is very similar to stain normalization,^36^^,^^37^ the focus is on translating one histological stain into another. For the purpose of domain adaptation, the images are virtually restained before processing. This approach is based on the assumption that annotated training data are only available for one particular stain that is approached by translating each stain to the target stain.

Similarly, Xu et al.^58^ adapted cycleGAN to translate between different stains (H&E and immunohistochemistry [IHC]) through the addition of a structured loss, aimed at suppressing bias (here referred to as “imaginary” features). Contrary to Gadermayr et al.,^64^ the authors did not apply further image analysis, such as classification or segmentation, but instead evaluated the realism of the data through Turing tests. They identified a large set of application fields including a fast and low-cost generation of IHC stainings, virtual multiplexing, co-localization, augmentation, and color deconvolution.

In a similar manner, an approach was proposed to translate between H&E and immunofluorescent stains.^40^ Unlike the approaches mentioned before,^58^^,^^64^ based on unpaired training (cycleGAN), here Pix2Pix (Figure 1E) was employed. Typically, paired samples showing exactly the same tissue in two different stains are difficult to collect. The authors, however, used multiplexed imaging, which allows for the generation of perfectly corresponding image pairs. Even though multiplexing could also be applied clinically, it is costly and can degrade both tissue quality and antigenicity. This kind of I2I translation might omit the need for clinical multiplexing.

Another Pix2Pix approach by Rana et al.^65^ similarly utilized I2I translation based on paired training to convert H&E to unstained image data (and vice versa). Pairs are available, since the unstained sample is captured before applying the H&E staining. Only additional registration is needed to obtain the pixel correspondences. Both approaches^40^^,^^65^ were qualitatively shown to successfully use Pix2Pix as an I2I translation model but, since they lack an evaluation in the form of subsequent classification or segmentation, a quantitative conclusion cannot be drawn.

Additionally, the Siamese GAN architecture introduced in ^34^ (Figure 1G) can be used for domain adaptation. This domain adaptation pipeline maps the source domain to the target domain in an unsupervised, feature-based (not image-based) manner. By combining adversarial and Siamese training procedures, features from the source domain are mapped to the target domain while still being kept structurally similar to the source domain.

Further potential of GANs in a segmentation scenario is shown in Gupta et al.,^54^ where GANs were applied to “enrich” the image domain. Instead of increasing the number of samples, as performed in case of data augmentation, the information per image was enlarged. By performing image translation between a source and several target stains for each image, additional virtually restained images were created. This data were used to train and test a segmentation network, which outperformed the baseline setting of a network trained and tested on the source domain only. However, a positive effect of this application was only shown in one very specific setting in the field of kidney pathology.

### Segmentation with Supervised Models

The Pix2Pix network is an established powerful segmentation approach exhibiting an alternative to conventional, fully convolutional segmentation networks.^27^ Wang et al.^59^ adapted the Pix2Pix model (Figure 1E) for the field of histology and showed improved performance compared with a stand-alone fully convolutional network.^66^ For many tasks, stand-alone fully convolutional networks^11^^,^^12^^,^^27^^,^^28^ (without an adversarial loss) perform reasonably well in histopathological image analysis. However, a difficulty here is the fine structure of the basal membrane. The GAN's advantage over these basic (non-adversarial) CNN approaches is given by the ability to maintain fine details through the adversarial loss.^42^

### Synthesis Enabling Weakly Supervised and Unsupervised Learning

One way to synthesize histological samples starts with the generation of label masks. Realistic shapes are obtained by sampling the parameters of the objects randomly from distributions corresponding to natural occurrences. Mahmood et al.^6^ directly used binary masks and added realism by deploying a generator of a cycle-GAN model. This model was trained on an unpaired dataset consisting of a label-domain and an image-domain dataset. Bug et al.^8^ proposed a similar approach. Instead of converting binary masks and images, the authors already added a certain kind of realism before GAN-based image translation. Specifically, they added typical colors as well as spot-noise combined with blur in the background as well as the foreground. Hou et al.^33^^,^^55^ placed even more focus on hand-crafted synthesis. Nucleus shapes were obtained by simulating roundish polygons. Background texture was obtained from original images by making use of unsupervised segmentation techniques. In a similar way, object texture was obtained from real objects. Background and foreground were then combined and an encoder-decoder GAN (Figure 1D) was trained to translate from the pre-computed synthetic domain to the real domain. Instead of the cycleGAN architecture, this encoder-decoder GAN with one generator and one discriminator was utilized, containing a regularization loss (*L*_1_ and *L*_2_ norm between the input and output image), a discriminator loss, and a task-specific loss,^67^^,^^68^ to focus on the generation of challenging samples.

Senaras et al.^69^ utilized a similar architecture (based on Pix2Pix) to generate realistic histopathological images from ground-truth label-mask images. The method is similar to that of Hou et al.,^33^^,^^55^ using an *L*_1_ and a discriminator loss. Compared with the other approaches,^6^^,^^8^^,^^33^^,^^55^ the goal here was not the (unsupervised) segmentation but the generation of a dataset to be used for the analysis of computer-based algorithms as well as inter- and intra-observer variability. Instead of artificially generated label masks, the authors translated ground-truth annotations into realistic images. The obtained pairs were intended to show perfect correspondence, which is not the case if data are annotated manually in the traditional sense.

A vice versa approach (in comparison with other methods^6^^,^^8^^,^^33^^,^^55^) was proposed by Gadermayr et al.^31^^,^^53^ Instead of generating virtual images out of label masks for means of obtaining labeled training samples, the authors performed translation directly from the image to the label-mask domain. Similar to Mahmood et al.,^6^ a cycleGAN model was trained to translate from the label-mask to the image domain and vice versa. The label masks were obtained by randomly sampling non-overlapping ellipses by varying rotation, aspect ratio, and size. Ultimately, the authors used the generator translating images to label and thereby immediately obtain segmentation output, circumventing the need for an additional segmentation model.

This translation between the image and the segmentation label-mask domain (and vice versa) exhibits a highly interesting field with the potential for unsupervised segmentation. The unpaired and, thereby, unsupervised approach can be combined with manually labeled data to improve performance even further (depending on the amount of labeling resources). Based on the publications so far, it is difficult to make a general statement as to whether a translation from the label-mask to the image domain or vice versa is more effective when compared with unsupervised segmentation. Performing a translation from the image to the label-mask domain is probably a task that is easier to learn. Gadermayr et al.^31^^,^^53^ were unable to generate realistic images, whereas the translation from the image to the label-mask domain showed a reasonable outcome.

A limitation is apparent because the mapping from the image to the label mask is more or less defined while the reversed mapping is ambiguous (also referred to as ill-posed or one-to-many mapping). This means that for one label mask there exist several corresponding images. This is quite obvious, since high-level label masks (e.g., stroma versus tumor) do not provide information on the placement of low-level features such as nuclei. In case of cycleGAN, both mappings (i.e., both generators) need to be trained independent of the finally needed generator. The “ambiguous” mapping, however, can affect training based on the cycle-consistency loss, as the loss ||*x*_1_−$G_{X_{1}}$∘$G_{X_{2}}$(*x*_1_)||_2_ is not necessarily small, even if the generators show attendant behavior. If the mapping represented by the generator $G_{X_{1}}$ is ambiguous, a variety of realistic images can be generated out of $G_{X_{2}}$. For a detailed discussion, we refer the reader to Gadermayr et al.^70^ Besides an explanation and discussion of the problem, this paper contains a simple yet effective method of resolution for one-to-many mappings. The problem is bypassed by removing half of the cycle-consistency loss. Other approaches are based on auxiliary latent spaces to control the variations of the one-to-many (or even many-to-many) mappings.^60^^,^^61^ The idea of these approaches is to decompose an image into a content code that is domain invariant and a domain code that captures domain-specific properties. The problem of ambiguous mapping is not limited to pathology but is a common problem in generic I2I settings. Another typical example exhibiting ambiguous mappings in medicine is magnetic resonance imaging-to-computed tomography synthesis.^71^, ^72^, ^73^

Another approach to unsupervised or weakly supervised learning is given by representation learning. Hu et al.^50^ adapted the GAN architecture for learning cell-level image representations in an unsupervised manner. For that purpose an auxiliary network was employed, which shares weights with the discriminator. In addition to the discriminator loss, the authors introduced a further mutual information loss. The trained auxiliary network can be employed to extract features on the cell level, which are used to perform cluster analysis. The authors utilized the aggregated cluster information to train an image-level classification model. However, these extracted features could also be applied for high-level image segmentation.

### Data Generation and Augmentation

Similar to work on domain adaptation,^64^ Wei et al.^29^ adapted the cycleGAN architecture to perform data augmentation. Instead of performing what we typically refer to as domain adaptation (i.e., the adjustment between slightly dissimilar distributions while the class labels remain similar), they trained the GAN architecture to translate from one tissue category to another (here from normal to abnormal). Thereby, they obtained a generation model for the means of data augmentation which creates, based on existing samples, additional samples of the other class. The difficulty of this task is that not only low-level image details, such as color, need to be changed. In contrast, a translation from one class to another typically requires a major change of the image morphology. The authors showed that this is effective in the considered application scenario,^29^ as the achieved classification performance could be increased. However, the cycleGAN architecture in general is not optimized for performing morphological changes. Similarly, as discussed in the previous subsection, the problem of ambiguous mapping emerges. The problem in the case of synthesis is that one mapping (from the label to the image domain) is ambiguous. Here, both mappings are potentially ambiguous, because there is typically not a one-to-many, and definitely not a one-to-one, mapping corresponding to pathological changes. Another approach for data generation was proposed in Quiros et al.,^38^ which focuses on generating artificial cancer tissue from a structured latent space using pathologyGAN (Figure 1B). The robustness of these data-augmentation methods was shown for many medical image analysis applications such as the generation of X-ray bone lesion images^74^ and images showing optical skin lesions.^56^

## Discussion: Potential of GANs

We identified three fields in digital pathology with a particularly high potential of GANs. These fields have been identified based on the related work summarized in the previous section. Certainly we do not claim the exclusive truth. On the contrary, we invite the reader to take a critical look at this review and expand on it in future research.

### Synthesis instead of Labeling

Firstly, we assessed the capability of cycleGAN and derivatives to translate from an image to a label domain (and vice versa) as an extremely powerful approach. The ability to learn from unpaired data in this setting translates a potentially time-consuming and cumbersome labeling problem into a synthesizing problem. For many applications in the field of digital pathology, synthesis of realistic ground-truth label maps is a feasible task. This is particularly the case as far as roundish-shaped objects that require a basic simulation model relying on only a handful of parameters are concerned.^6^^,^^8^^,^^53^

A difficulty (as discussed earlier) is that the mapping from the label to the image domain is mostly ambiguous, as a label mask can be mapped to more than one corresponding image. This potentially complicates training of the GAN architecture with diverse methods of resolution.

One approach to tackle this challenge is to change the cycleGAN architecture,^60^^,^^61^^,^^70^ as discussed in the subsection Synthesis Enabling Weakly Supervised and Unsupervised Learning. Another method of resolution is outlined in Gadermayr et al.^31^ The authors showed that cycleGAN training is clearly more effective when the label-mask domain contains additional information on the corresponding image context. A synthesis of low-level information (nuclei) in addition to the high-level objects (which need to be segmented) clearly improved overall performance and robustness. The additional information was stored in a separate image channel. An improvement is obtained because the generator networks now receive information on where to place the low-level objects in order to obtain a low cycle-consistency loss. The difficulty here is that the simulation model thereby becomes more complex. However, especially in case of high-level objects with complex shapes, we are confident that this is the most powerful solution when unpaired approaches, such as cycleGAN, should be trained to perform translations from a label mask to an image domain or vice versa.

For practical reasons, an adjustable simulation tool would be very helpful to easily and quickly generate simulated label-mask data according to the characteristics of an individual task. As segmentation tasks in digital pathology often correspond to rather uniform roundish objects, basic functionality would be sufficient for many purposes.

So far, these unpaired approaches have not been applied and adapted to applications without strong shape constraints, as, for example, in tumor segmentation.^13^ Apart from the very diverse morphology of the individual regions, a difficulty here arises in the scale of the regions of interest. Regions can show up to several hundreds or even thousands of pixels in diameter. As the segmentation networks are applied patchwise, this can constitute a challenge. A method of resolution might be a multi-level (or multi-resolution) approach. Small, morphologically regular structures (such as nuclei) could be extracted in a first step. Afterward, a segmentation of high-level objects (such as stroma, tumor) can be performed at a lower resolution, based on the segmentation information extracted at the lower level.

### Potential of Stain-to-Stain Translation

We further identified stain-to-stain translation as an application with high potential. In previous work, stain-to-stain translation was performed mostly for the compensation of domain shifts between training and test data. However, previous work also showed that a GAN is capable of facilitating a segmentation task by either changing the appearance or by adding information to the image domain.^54^^,^^64^ This is remarkable, as all additional information is extracted from the original images. This raises the question as to why processing in two steps (translation followed by segmentation) can be more effective than direct segmentation. We assume that there are stains which are easier to process (segment) than others, and that a separation of a problem into two easier tasks can be beneficial. In this way, the individual networks can fully focus on the individual tasks that are quite different. While stain translation requires rather little context, a segmentation task (of high-level objects) surely requires a large context. To further exploit this effect, special stains could be applied or developed that would particularly highlight the respective objects of interest to increase the performance. Another option is the translation from bright-field to fluorescence microscopy,^40^ which has the potential of trivializing the subsequent segmentation task.

A question that has not been addressed so far is whether a translation from a stain to another (e.g., a general-purpose stain, such as H&E, to an IHC stain) is capable of showing features similar to those of the real target stain. Modanwal et al.^64^ performed such a translation but only for segmenting higher-level objects, which are independent of the histological stain. The requirement here was only that the morphology of the (high-level) objects of interest is maintained. To show whether or not GANs are able to generate virtual stains that are not only realistic but also exhibit features on a low level (i.e., the stain response on pixel level) similar to those of a real stain, a special dataset is needed. This requires a large dataset containing corresponding slide pairs stained with two different approaches. Apart from the capabilities of the neural networks, the principal question here is whether the information is available in the image data or not, which might differ from problem to problem. In any event, due to the immense impact we are confident that this is worth studying.

### Morphology Translation

I2I translation typically covers mappings from one domain to another, where the domain gap is caused by (intentional or unintentional) variability in the data-generation protocol.^35^^,^^36^^,^^40^^,^^58^^,^^64^^,^^65^ In each of these settings, color and potentially also texture varies between the domains. However, as the underlying tissue is unchanged, there are mostly no clear morphological changes.

A setting with morphological changes has been investigated by Wei et al.^29^ The authors of this paper explored a translation between data from different classes for the means of data augmentation. They used a derivation of the cycleGAN architecture and achieved improvements regarding the final classification task, but also figured out that there is a clear difference between the generated and the real image data. This statement was reinforced by the finally obtained classification rates, which were clearly higher for the real than for virtual data.

Even though cycleGAN generally shows high versatility, we are confident that it does not exhibit the optimum architecture for settings with changed morphology. In the case of morphological changes, there occur typically ambiguous mappings that can be highly problematic (as already discussed in Synthesis Enabling Weakly Supervised and Unsupervised Learning).

Furthermore, conventional CNNs, used as generator models, are not optimally suited to perform spatial translation.^75^ Consequently, we are confident that this field exhibits potential for further improvements with specifically optimized architectures. Methods including a spatial transformer module^75^ might be beneficial for this purpose. A powerful tool could facilitate a translation between healthy and pathological data, for example, for the means of data augmentation. In addition, a translation between different imaging settings (such as frozen-to-paraffin translation) might be considered, potentially improving the image quality and also, therefore, the final classification accuracy.

### Conclusion

In this paper, we have summarized existing GAN architectures in the field of histological image analysis. We have provided an overview of addressed application scenarios and the methods employed and have identified the major fields of research. Apart from current trends and benefits of GANs, we have also identified the remaining potential and the appeal for novel technical approaches to improve image analysis even further. In general, it can be stated that GANs exhibit the potential for relaxation or even elimination of the constraint upon the large amounts of annotated training data required to train deep neural network architectures. Despite the remaining challenges, we consider that this technology will play a key role in the practical application of flexible image analysis methods in digital pathology.
