# Generative Adversarial Networks in Digital Pathology: A Survey on Trends and Future Potential

**DOI:** 10.1016/j.patter.2020.100144

**Published:** 2020-11-13

**Authors:** Maximilian E. Tschuchnig, Gertie J. Oostingh, Michael Gadermayr

## Main Text

(PATTER *1*, 100089-1–100089-11; September 11, 2020)

Due to an error in production, the following equation had a typographical error. The equation was published as (*MI*(*M*_11_ (x_1*i*_ ),*M*_12_ (x_*ij*_))) and the correct equation should be (*MI*(*M*_11_(x_1*i*_),*M*_12_(x_1*j*_))). This change does not affect the conclusions of the paper and has been corrected online. We sincerely apologize to the readers for any confusion that may have resulted from these errors.

